# Malnutrition determined by the controlling nutritional status score, prognostic nutritional index and hemoglobin-albumin-lymphocyte-platelet score is associated with prolonged hospital stay in liver cancer patients who underwent interventional treatment

**DOI:** 10.3389/fonc.2026.1723228

**Published:** 2026-01-30

**Authors:** Zhanshang Zhang, Lian Liao, Yuanbiao Zeng, Peng Ouyang

**Affiliations:** Department of Interventional Therapy, Meizhou People’s Hospital, Meizhou, China

**Keywords:** controlled nutritional status, hemoglobin-albumin-lymphocyte-platelet, interventional treatment, length of hospital stay, liver cancer, prognostic nutritional index

## Abstract

**Background:**

The purpose of this study was to investigate the association between the nutritional status evaluated by the Controlling Nutritional Status (CONUT) score, Prognostic Nutritional Index (PNI) and hemoglobin-albumin-lymphocyte-platelet (HALP) score, and the prolonged hospital stay in liver cancer patients who underwent interventional treatment, in order to provide a reference for clinical optimization of the prognosis of liver cancer patients undergoing interventional treatment through nutritional assessment.

**Methods:**

466 liver cancer patients who underwent interventional treatment were retrospectively analyzed. Medical records (age, gender, history of smoking, history of alcohol drinking, hypertension, diabetes mellitus, viral hepatitis, and laboratory test results) were collected. The threshold for prolonged hospital stay was defined based on the third quartile (75th percentile) of length of hospital stay. The relationship between CONUT, PNI, and HALP and prolonged hospital stay was analyzed.

**Results:**

The mean hospital stay of patients was 11.0 (8.0, 14.0) days. There were 337 (72.3%) patients without prolonged hospital stay (<14.0 days) and 129 (27.7%) patients with prolonged hospital stay (≥14.0 days). The proportion of moderate and severe grade malnutrition determined by CONUT score in patients with prolonged hospital stay was higher than patients without prolonged hospital stay (*p=*0.002). The levels of PNI (*p=*0.002) and HALP (*p=*0.003) in patients with prolonged hospital stay were higher than those in patients without prolonged hospital stay. Logistic regression analysis showed that CONUT moderate + severe malnutrition grade (odds ratio (OR): 1.634, 95% confidence interval (CI): 1.067-2.503, *p* = 0.024), low PNI (OR: 1.684, 95% CI: 1.108-2.561, *p* = 0.015), and low HALP (OR: 1.666, 95% CI: 1.097-2.530, *p* = 0.017) were independently associated with prolonged hospital stay.

**Conclusions:**

Malnutrition defined by the CONUT, PNI, and HALP scores was significantly associated with the prolonged hospital stay in liver cancer patients who underwent interventional treatment.

## Introduction

Primary liver cancer is a type of malignant tumor that originates from liver cells or the epithelial cells of the intrahepatic bile ducts (1). It is characterized by a high degree of malignancy, rapid progression, and poor prognosis (2, 3). Based on the pathological histological features, primary liver cancer can be further classified into three subtypes: hepatocellular carcinoma (HCC) (4), cholangiocarcinoma (CCA) (5), and combined hepatocellular carcinoma-cholangiocarcinoma (3). And HCC accounts for the largest proportion, accounting for approximately 70% to 90% of all primary liver cancers (6). In recent years, the number of new cases and deaths from liver cancer worldwide has consistently ranked among the top five for all malignant tumors in terms of incidence and mortality (7, 8).

At present, based on factors such as the patient’s tumor stage, liver function status, and overall physical condition, liver cancer can be treated through various methods including surgery (9), ablation therapy (10), targeted therapy, immunotherapy (11), and interventional therapy (12). Transcatheter Arterial Chemoembolization (TACE) is currently the standard treatment for patients with intermediate-stage liver cancer, and it can also be used for palliative treatment for some patients with advanced liver cancer (13, 14). The therapeutic principle is to inject chemotherapy drugs and embolic agents into the blood supply arteries of the tumor through a catheter (15). The embolic agent will block the blood supply to the tumor, causing it to suffer from ischemia and hypoxia (16). Meanwhile, the chemotherapy drugs directly act on the tumor cells, exerting an anti-tumor effect (17). Clinical practice has shown that there are significant individual differences in the recovery process of liver cancer patients after interventional treatment (18). Some patients require an extended hospital stay due to slow recovery, which not only increases the consumption of medical resources but may also affect the subsequent treatment connection and quality of life of the patients.

In-depth investigation into the influencing factors of prolonged hospital stay after interventional treatment is of great significance for optimizing clinical management strategies. The incidence of malnutrition among liver cancer patients is relatively high, and its causes are multi-dimensional and complex (19). The rapid proliferation of tumor cells will extensively seize the body’s nutrients, leading to imbalance in energy and protein metabolism (20). Liver function impairment directly affects the digestion, absorption, and synthesis functions of nutrients, such as reduced albumin synthesis and abnormal bilirubin metabolism (21). The malnutrition form a vicious cycle of nutritional deterioration - decreased treatment tolerance - delayed recovery, which not only reduces the patient’s tolerance to interventional treatment but also increases the risks of postoperative infections and ascites, ultimately delaying the recovery process.

Nutritional status is a key determinant of disease prognosis, with malnourished populations exhibiting heightened disease susceptibility and an elevated risk of adverse outcomes (22). Currently, clinically validated nutritional evaluation instruments primarily include the Subjective Global Assessment (SGA) (23), and the Nutritional Risk Screening 2002 (NRS 2002) (24). However, SGA relies heavily on subjective clinical judgments, leading to potential inter-observer variability, while NRS-2002 requires integration of multiple complex clinical parameters (e.g., weight loss, disease severity) that may reduce its efficiency in busy clinical settings or for patients with incomplete medical data. Additionally, neither tool is specifically optimized for special patient populations (such as those undergoing interventional therapy or with chronic liver disease), which may compromise the accuracy and applicability of nutritional risk stratification in these groups.

Assessing the nutritional status plays a crucial role in the prognosis evaluation of patients (22). The Prognostic Nutritional Index (PNI) comprehensively reflects the nutritional and immune status through the combination of albumin levels and lymphocyte counts, and has been proven in multiple studies to be an independent predictor of survival prognosis for liver cancer patients (25). The Control Nutritional Status (CONUT) score integrates serum albumin, total cholesterol, and lymphocyte counts, which can effectively assess the body’s nutritional reserves and immune function (26). The Hemoglobin - Albumin - Lymphocyte - Platelet (HALP) score combines four basic indicators to balance the assessment of hematopoietic function and nutritional status, providing support for multi-dimensional nutritional judgment (27). These quantitative scoring tools based on objective laboratory indicators all have the advantages of easy data acquisition and objective results, and show good application prospects.

Although studies have confirmed that malnutrition is closely related to the prognosis of liver cancer patients and that nutritional support therapy can reduce the risk of complications and shorten hospital stay, studies on the specific association between the nutritional status defined by the CONUT, PNI, and HALP scoring tools and the prolonged hospital stay after liver cancer intervention treatment are still relatively scarce. Clarifying the relationship between the three nutritional status scores and the length of hospitalization regarding the nutritional deficiency conditions is of great significance for assessing the prognosis of patients and optimizing treatment plans. This study aims to explore the relationship between nutritional quantitative indicators and the prolonged hospital stay in liver cancer patients who underwent interventional treatment.

## Materials and methods

### Study cohort

This study is a retrospective study. The research subjects were selected from liver cancer patients who underwent interventional treatment at Meizhou People’s Hospital, from December 2019 to March 2024. All the patients’ medical data were obtained from the hospital’s electronic medical record system, including the first page of the hospitalization medical record, laboratory test reports, imaging examination materials, surgical records, and so on.

Inclusion criteria: (1) met the diagnostic criteria for primary liver cancer, and be confirmed as having primary liver cancer through pathological examination, enhanced CT or MRI imaging tests (28); (2) patients who received interventional therapy, whose interventional treatment regimens conformed to clinical diagnostic and therapeutic criteria, and who had not received nutritional support prior to treatment; (3) age ≥ 18 years old, gender not limited; (4) complete clinical data, including laboratory test indicators before interventional treatment and relevant records of hospitalization time; and (5) the patients have given informed consent.

Exclusion criteria as follows: (1) concurrented with other malignant tumors or metastatic liver cancer; (2) before the interventional treatment, there were severe infections, liver failure, severe coagulation disorders or other serious systemic diseases (such as cardiac insufficiency, and renal failure); (3) presence of hematological diseases or recent receipt of treatments such as radiotherapy, chemotherapy, and immunotherapy that may affect nutritional and routine blood test indices; (4) during the interventional treatment, serious complications occurred and required emergency surgical intervention; (5) key indicators in the clinical data are missing; and (6) due to non-treatment-related reasons (such as family requests for early discharge, and transfer for treatment) the recorded hospitalization time is inaccurate. A total of 466 patients with liver cancer were finally included in this study for investigation. This study was approved by the Human Ethics Committees of the Meizhou *People’s Hospit*al.

### Evaluation of nutritional status

The nutritional status of all patients was evaluated using the Control Nutritional Status (CONUT) score, prognostic nutritional index (PNI), and the Hemoglobin - Albumin - Lymphocyte - Platelet (HALP) score.

Scoring criteria of CONUT: (1) serum albumin concentration: ≥35 g/L (0 point), 30-34.9 g/L (2 points), 25-29.9 g/L (4 points), and <25 g/L (6 points); (2) peripheral lymphocyte count: ≥1.6 × 10^9^ count/L (0 point), 1.2-1.59 × 10^9^ count/L (1 point), 0.8-1.19 × 10^9^ count/L (2 points), and <0.8 × 10^9^ count/L (3 points); and (3) total cholesterol: >180 mg/dL (0 point), 140–180 mg/dL (1 point), ≥100 and <140 mg/dL (2 points), and <100 mg/dL (3 points). CONUT score is the sum of the scores of serum albumin, lymphocyte count, and total cholesterol level. The malnutrition grades of the CONUT score: normal (score 0-1), light (score 2-4), moderate (score 5-8), and severe (score 9-12) (29).

The PNI score is calculated based on the serum albumin level (g/L) and the peripheral blood lymphocyte count (×10^9^/L). The calculation formula is as follows: PNI=serum albumin (g/L) + 5 × lymphocyte count (×10^9^/L).

HALP score is based on hemoglobin (g/L), serum albumin (g/L), lymphocyte count (×10^9^/L) and platelet count (×10^9^/L), and the calculation formula is: HALP = hemoglobin × albumin × lymphocyte count/platelet count.

### Data collection

The clinical data were collected, including age, gender, history of smoking, history of alcohol drinking, hypertension, diabetes mellitus, viral hepatitis, and laboratory test results (serum albumin, peripheral lymphocyte count, total cholesterol, hemoglobin, and platelet count). Laboratory test results were collected during the first hospital examination. The threshold for prolonged hospital stay was defined based on the third quartile (75th percentile) of the length of hospital stay in all patients.

### Statistical analysis

Data analysis was performed using SPSS statistical software version 26.0 (IBM Inc., USA). Comparisons among variables that follow a normal distribution were analyzed using independent sample t-test. Variables that do not follow a normal distribution are conducted using Mann-Whitney U test for groups comparisons. Categorical variables were expressed as the number of cases (%), and compared between groups using the χ^2^ test. The specificity and sensitivity of PNI and HALP were described using the receiver operating characteristic (ROC) curve analysis. The accuracy of PNI and HALP in differentiating prolonged and non-prolonged hospital stay was evaluated by calculating the area under the ROC curve (AUC), and the optimal cut-off values of PNI and HALP were determined using the Youden index. Logistic regression analysis was used to analysis the relationship of CONUT, PNI, HALP and prolonged hospital stay adjusting for influencing factors (age, gender, history of smoking, history of alcohol drinking, hypertension, diabetes mellitus, and viral hepatitis). *p* < 0.05.

## Results

### The clinical features in patients with liver cancer who have undergone interventional treatment

There were 406 (87.1%) and 60 (12.9%) male and female patients, and the mean age of the patients was 61.5 (53.8, 70.0). There were 89 (19.1%), 62 (13.3%), 120 (25.8%), 89 (19.1%), and 365 (78.3%) patients had history of smoking, history of alcohol drinking, hypertension, diabetes mellitus, and viral hepatitis (**Table 1**).

**Table 1:** The clinical features in patients with liver cancer who have undergone interventional treatment.

Clinical characteristics	Total (n=466)
Age (years), median (IQR)	61.5 (53.8, 70.0)
Gender	
Male, n(%)	406 (87.1%)
Female, n(%)	60 (12.9%)
History of smoking	
No, n(%)	377 (80.9%)
Yes, n(%)	89 (19.1%)
History of alcohol drinking	
No, n(%)	404 (86.7%)
Yes, n(%)	62 (13.3%)
Hypertension	
No, n(%)	346 (74.2%)
Yes, n(%)	120 (25.8%)
Diabetes mellitus	
No, n(%)	377 (80.9%)
Yes, n(%)	89 (19.1%)
Viral hepatitis	
No, n (%)	101 (21.7%)
Yes, n (%)	365 (78.3%)
Hospital stay (days), median (IQR)	11.0 (8.0, 14.0)

IQR, interquartile range.

### Comparison of the clinical characteristics of patients with and without prolonged ICU stay

In this study, the mean hospital stay of patients was 11.0 (8.0, 14.0) days. The threshold for prolonged hospital stay was defined as ≥14.0 days. There were 337 (72.3%) patients without prolonged hospital stay (<14.0 days) and 129 (27.7%) patients with prolonged hospital stay (≥14.0 days).

The proportion of moderate and severe grade malnutrition determined by the CONUT score in patients with prolonged hospital stay was higher than patients without prolonged hospital stay (*p=*0.002, χ^2^ = 14.450). The levels of PNI (*p=*0.002, Z=-3.171) and HALP (*p=*0.003, Z=-2.951) in patients with prolonged hospital stay were higher than those in patients without prolonged hospital stay. There was no statistically significant difference in age, gender, and proportion of history of smoking, alcohol drinking, hypertension, diabetes mellitus, and viral hepatitis between the two groups (**Table 2**).

**Table 2:** Comparison of the clinical characteristics of patients with and without prolonged hospital stay in patients with liver cancer who have undergone interventional treatment.

Clinical characteristics	Non-prolonged hospital stay (n=337)	Prolonged hospital stay (n=129)	*P* (χ^2^/Z)
Age (years), median (IQR)	61.0 (53.5, 70.0)	63.0 (53.5, 71.0)	0.531 (Z=-0.626)
Gender			
Male, n(%)	298 (88.4%)	108 (83.7%)	0.215 (χ^2^ = 1.842)
Female, n(%)	39 (11.6%)	21 (16.3%)	
History of smoking			
No, n(%)	276 (81.9%)	101 (78.3%)	0.429 (χ^2^ = 0.784)
Yes, n(%)	61 (18.1%)	28 (21.7%)	
History of alcohol drinking			
No, n(%)	294 (87.2%)	110 (85.3%)	0.648 (χ^2^ = 0.314)
Yes, n(%)	43 (12.8%)	19 (14.7%)	
Hypertension			
No, n(%)	253 (75.1%)	93 (72.1%)	0.554 (χ^2^ = 0.434)
Yes, n(%)	84 (24.9%)	36 (27.9%)	
Diabetes mellitus			
No, n(%)	275 (81.6%)	102 (79.1%)	0.598 (χ^2^ = 0.387)
Yes, n(%)	62 (18.4%)	27 (20.9%)	
Viral hepatitis			
No, n (%)	69 (20.5%)	32 (24.8%)	0.317 (χ^2^ = 1.031)
Yes, n (%)	268 (79.5%)	97 (75.2%)	
CONUT			
Normal, n(%)	77 (22.8%)	12 (9.3%)	0.002 (χ^2^ = 14.450)
Light, n(%)	151 (44.8%)	60 (46.5%)	
Moderate, n(%)	91 (27.0%)	43 (33.3%)	
Severe, n(%)	18 (5.3%)	14 (10.9%)	
PNI, median (IQR)	41.6 (36.8, 46.3)	39.4 (35.6, 43.2)	0.002 (Z=-3.171)
HALP, median (IQR)	33.6 (22.3, 52.5)	27.8 (15.8, 43.9)	0.003 (Z=-2.951)

CONUT, controlling nutritional status; PNI, prognostic nutritional index; HALP, hemoglobin-albumin-lymphocyte-platelet score; IQR, interquartile range.

### Comparison of the clinical characteristics of patients with and without prolonged hospital stay in patients with different nutritional statuses

**Figure 1:**
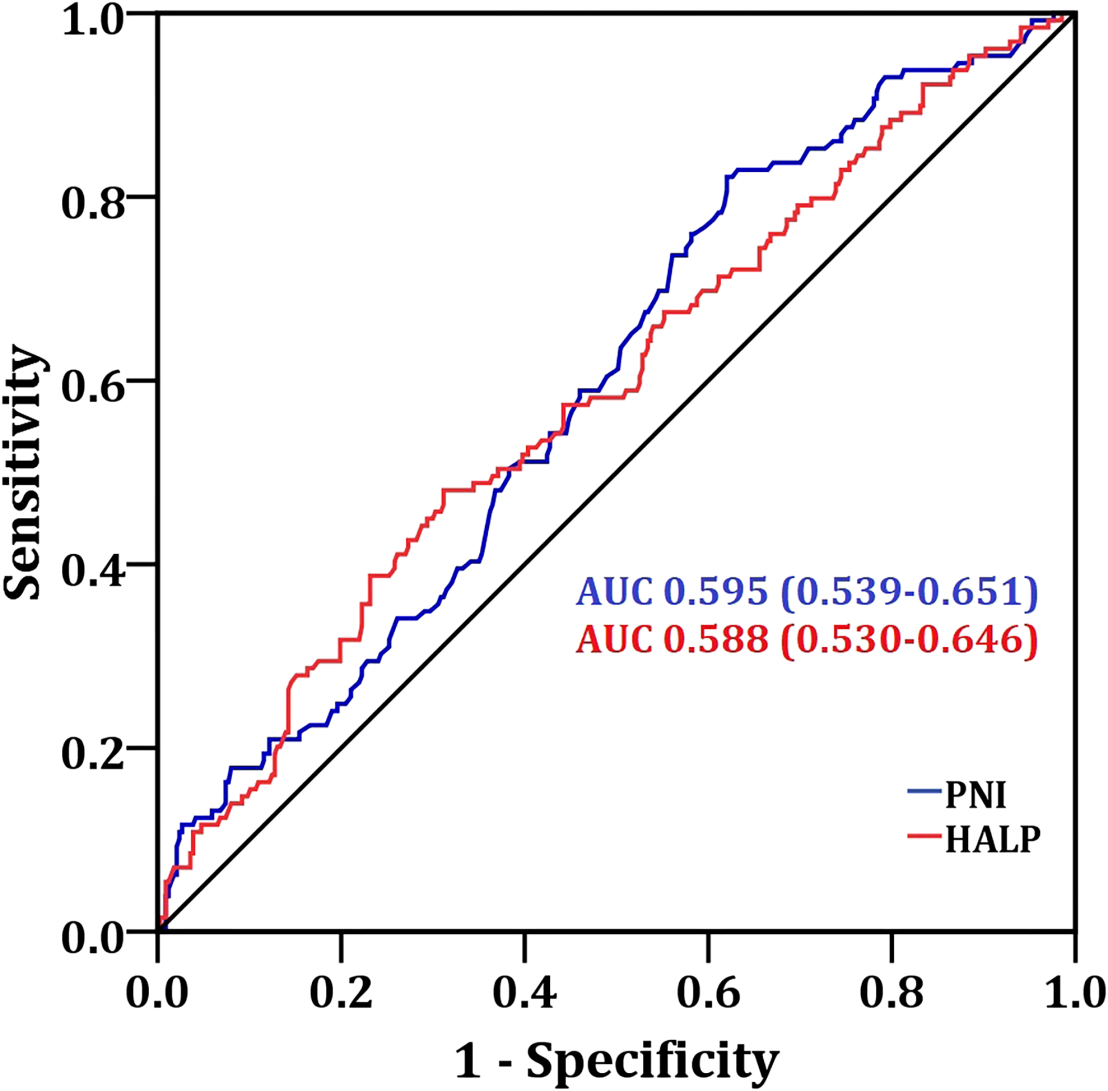
ROC analysis of PNI and HALP used in the prolonged hospital stay in patients with liver cancer who have undergone interventional treatment. PNI, prognostic nutritional index; HALP, hemoglobin-albumin-lymphocyte-platelet score; AUC, area under receiver operating characteristic curve.

The cutoff value of PNI was 40.85 (sensitivity=58.9%, specificity=54%, AUC = 0.595), and the HALP score cutoff value was 31.9 (sensitivity=57.4%, specificity=55.5%, AUC = 0.588) by ROC curve analysis (**Figure 1**).

The proportions of patients with prolonged hospital stay (34.3% vs. 24.0%, *p* = 0.018, χ^2^ = 5.704) in patients with moderate/severe malnutrition based on CONUT score (n=166) were higher than those in patients with normal/light malnutrition based on CONUT score (n=300). The proportion of prolonged hospital stay (32.9% vs. 22.6%, *p* = 0.013, χ^2^ = 6.230) in patients with low PNI score (<40.85) (n=231) was higher than that in patients with high PNI (≥40.85) (n=235). The proportion of prolonged hospital stay (33.0% vs. 22.7%, *p* = 0.017, χ^2^ = 6.175) in patients with low HALP score (<31.9) (n=224) was higher than that in patients with high HALP (≥31.9) (n=242) (**Table 3**).

**Table 3:** Comparison of the clinical characteristics of patients with and without prolonged hospital stay in patients with different nutritional statuses.

Clinical characteristics	Normal + light malnutrition based on CONUT score (n=300)	Moderate + severe malnutrition based on CONUT score (n=166)	*P* (χ^2^)	Low PNI (<40.85) (n=231)	High PNI (≥40.85) (n=235)	*P* (χ^2^)	Low HALP (<31.9) (n=224)	High HALP (≥31.9) (n=242)	*P* (χ^2^)
Age (years), median (IQR)	59.5 (53.0, 68.0)	64.0 (56.0, 72.0)	0.001 (Z=-3.362)	64.0 (56.0, 72.0)	60.0 (52.0, 67.0)	0.001 (Z=-3.205)	63.0 (53.0, 72.0)	60.0 (54.0, 69.0)	0.500 (Z=-0.674)
Gender									
Male, n(%)	266 (88.7%)	140 (84.3%)	0.195 (χ^2^ = 1.786)	196 (84.8%)	210 (89.4%)	0.167 (χ^2^ = 2.115)	190 (84.8%)	216 (89.3%)	0.168 (χ^2^ = 2.039)
Female, n(%)	34 (11.3%)	26 (15.7%)		35 (15.2%)	25 (10.6%)		34 (15.2%)	26 (10.7%)	
History of smoking									
No, n(%)	241 (80.3%)	136 (81.9%)	0.713 (χ^2^ = 0.176)	190 (82.3%)	187 (79.6%)	0.481 (χ^2^ = 0.540)	181 (80.8%)	196 (81.0%)	1.000 (χ^2^ = 0.003)
Yes, n(%)	59 (19.7%)	30 (18.1%)		41 (17.7%)	48 (20.4%)		43 (19.2%)	46 (19.0%)	
History of alcohol drinking									
No, n(%)	257 (85.7%)	147 (88.6%)	0.398 (χ^2^ = 0.773)	204 (88.3%)	200 (85.1%)	0.341 (χ^2^ = 1.038)	190 (84.8%)	214 (88.4%)	0.276 (χ^2^ = 1.313)
Yes, n(%)	43 (14.3%)	19 (11.4%)		27 (11.7%)	35 (14.9%)		34 (15.2%)	28 (11.6%)	
Hypertension									
No, n(%)	223 (74.3%)	123 (74.1%)	1.000 (χ^2^ = 0.003)	174 (75.3%)	172 (73.2%)	0.672 (χ^2^ = 0.277)	170 (75.9%)	176 (72.7%)	0.459 (χ^2^ = 0.610)
Yes, n(%)	77 (25.7%)	43 (25.9%)		57 (24.7%)	63 (26.8%)		54 (24.1%)	66 (27.3%)	
Diabetes mellitus									
No, n(%)	246 (82.0%)	131 (78.9%)	0.461 (χ^2^ = 0.658)	189 (81.8%)	188 (80.0%)	0.639 (χ^2^ = 0.249)	192 (85.7%)	185 (76.4%)	0.013 (χ^2^ = 6.467)
Yes, n(%)	54 (18.0%)	35 (21.1%)		42 (18.2%)	47 (20.0%)		32 (14.3%)	57 (23.6%)	
Viral hepatitis									
No, n (%)	67 (22.3%)	34 (20.5%)	0.725 (χ^2^ = 0.216)	47 (20.3%)	54 (23.0%)	0.502 (χ^2^ = 0.476)	54 (24.1%)	47 (19.4%)	0.260 (χ^2^ = 1.504)
Yes, n (%)	233 (77.7%)	132 (79.5%)		184 (79.7%)	181 (77.0%)		170 (75.9%)	195 (80.6%)	
Prolonged hospital stay									
No, n (%)	228 (76.0%)	109 (65.7%)	0.018 (χ^2^ = 5.704)	155 (67.1%)	182 (77.4%)	0.013 (χ^2^ = 6.230)	150 (67.0%)	187 (77.3%)	0.017 (χ^2^ = 6.175)
Yes, n (%)	72 (24.0%)	57 (34.3%)		76 (32.9%)	53 (22.6%)		74 (33.0%)	55 (22.7%)	

CONUT, controlling nutritional status; PNI, prognostic nutritional index; HALP, hemoglobin-albumin-lymphocyte-platelet score.

### Logistic regression analysis of associated factors with prolonged hospital stay

The results of univariate analysis indicated that CONUT moderate + severe malnutrition grade (odds ratio (OR): 1.656, 95% confidence interval (CI): 1.093-2.510, *p* = 0.017), low PNI (OR: 1.684, 95% CI: 1.116-2.540, *p* = 0.013), and low HALP (OR: 1.677, 95% CI: 1.113-2.527, *p* = 0.013) were significantly associated with prolonged hospital stay (**Table 4**).

**Table 4:** Logistic regression analysis of associated factors with prolonged hospital stay.

Variables	Crude β/OR (95% CI)	*P* values	Adjusted β/OR (95% CI)	*P* values
Prolonged ICU stay				
CONUT moderate + severe malnutrition	1.656 (1.093-2.510)	0.017	1.634 (1.067-2.503)	0.024
Low PNI	1.684 (1.116-2.540)	0.013	1.684 (1.108-2.561)	0.015
Low HALP	1.677 (1.113-2.527)	0.013	1.666 (1.097-2.530)	0.017
CONUT moderate + severe malnutrition or low PNI	1.698 (1.125-2.563)	0.012	1.699 (1.116-2.586)	0.013
CONUT moderate + severe malnutrition or low HALP	1.951 (1.255-3.032)	0.003	1.937 (1.231-3.048)	0.004
Low PNI or low HALP	1.787 (1.132-2.822)	0.013	1.756 (1.100-2.802)	0.018
CONUT moderate + severe malnutrition plus low PNI	1.649 (1.087-2.502)	0.019	1.626 (1.060-2.493)	0.026
CONUT moderate + severe malnutrition plus low HALP	1.683 (1.059-2.675)	0.028	1.661 (1.038-2.658)	0.034
Low PNI plus low HALP	1.830 (1.197-2.797)	0.005	1.845 (1.200-2.837)	0.005
CONUT moderate + severe malnutrition or low PNI or low HALP	1.765 (1.118-2.786)	0.015	1.736 (1.087-2.772)	0.021
CONUT moderate + severe malnutrition plus low PNI plus low HALP	1.651 (1.035-2.634)	0.035	1.632 (1.016-2.621)	0.043

CONUT, controlling nutritional status; OR, odds ratio; CI, confidence interval.

Adjust for: age, gender, history of smoking, history of alcohol drinking, hypertension, diabetes mellitus, and viral hepatitis.

In multivariate logistic regression analysis, CONUT moderate + severe malnutrition grade (OR: 1.634, 95% CI: 1.067-2.503, *p* = 0.024), low PNI (OR: 1.684, 95% CI: 1.108-2.561, *p* = 0.015), and low HALP (OR: 1.666, 95% CI: 1.097-2.530, *p* = 0.017) were independently associated with prolonged hospital stay adjusting for age, gender, history of smoking, history of alcohol drinking, hypertension, diabetes mellitus, and viral hepatitis (**Table 4**). In addition, we analyzed the relationship between different combinations of abnormal CONUT, PNI, and HALP indices and prolonged hospital stay. The results indicated that different combinations of abnormal CONUT, PNI and HALP indices were all risk factors for prolonged hospital stay in patients; however, no particularly significant differences were observed in the associations between these different combinations and prolonged hospital stay, while the CONUT moderate + severe malnutrition or low HALP group had the highest odds ratio (OR = 1.937) and the most significant *p* value (*p* = 0.004) (**Table 4**).

## Discussion

This study investigated the relationship between the nutritional status defined by the CONUT score, PNI score, and HALP score and the prolonged hospital stay of liver cancer patients after undergoing interventional treatment. The results showed that the malnutrition determined by all three scoring tools was significantly associated with the prolonged hospital stay of liver cancer patients after interventional treatment. Even after adjusting for other confounding factors, malnutrition remained an independent risk factor for the prolonged hospital stay. This finding provides a scientific basis for clinicians to assess the prognosis after interventional treatment by quantifying nutritional indicators.

The three nutritional status assessment tools reflect abnormal nutritional-immune-metabolic status of the body through different dimensions (30). Ultimately, they jointly lead to an increase in hospital stay by affecting the efficiency of liver function repair and the ability of tissue healing after surgery. The PNI score comprehensively assesses the body’s nutritional reserves and immune function through the synergistic effect of albumin and lymphocyte counts (31). The CONUT score is an extension of the PNI score, which includes the addition of the total cholesterol indicator. The CONUT score is based on serum albumin, total cholesterol, and lymphocyte count, which respectively correspond to the body’s protein reserves, energy metabolism, and cellular immune function (31).

Albumin is a key raw material for tissue repair. Its deficiency can lead to delayed repair of liver capsule damage and prolong the duration of postoperative abdominal pain (32, 33). Due to the damage of liver cells in patients with liver cancer, the synthesis ability of albumin has decreased. During interventional treatment, the blocking of hepatic artery blood supply by embolic agents will further aggravate the ischemia and hypoxia of liver cells, resulting in a continuous decrease in albumin levels (34). Total cholesterol is an important energy source for the body, and its level reflects the liver’s lipid metabolism function. In patients with liver cancer, the liver’s ability to synthesize and metabolize cholesterol declines (35). After interventional treatment, liver cell damage worsens, further inhibiting the lipid metabolism ability of liver cells. In a low-cholesterol state, liver cells have insufficient energy supply and cannot meet the energy consumption required for postoperative repair. At the same time, cholesterol is an important component of the cell membrane (36), and its lack will lead to a decrease in the stability of liver cell membranes, increasing the risk of liver cell necrosis, which may induce postoperative liver dysfunction and result in a poor prognosis. Lymphocyte count is a core indicator reflecting the cellular immune function (37). In patients with liver cancer, due to tumor consumption and abnormal liver function, the generation and activation of lymphocytes are hindered (38). The stress response triggered by interventional treatment will further lead to an increase in lymphocyte apoptosis. Lymphocyte reduction will significantly weaken the body’s ability to control local inflammation, and may result in an extended hospital stay.

Some studies found that the CONUT score was an independent prognostic indicator for the prognosis of hepatocellular carcinoma (HCC) patients (39, 40). The research conducted by Müller et al. revealed that PNI has predictive value for the overall survival (OS) of liver cancer patients undergoing transarterial chemoembolization (TACE), while the CONUT score does not have such an effect (41). However, some studies indicated that a high CONUT score was found to be a prognostic factor for poor OS in patients with hepatocellular carcinoma who received conventional transcatheter arterial chemoembolization (cTACE) treatment (42) and liver resection (43). In addition, some studies found that PNI was a prognostic parameter for HCC patients (25, 44, 45).

The HALP score integrates four indicators: hemoglobin, albumin, lymphocytes, and platelets (46). Its unique advantage lies in reflecting the common nutritional-hematopoietic dual abnormalities in patients with liver cancer (47). Hemoglobin is the key carrier for transporting oxygen. Patients with liver cancer have a tendency towards anemia due to chronic blood loss (such as minor bleeding from esophageal and gastric varices) and reduced synthesis of erythropoietin (the liver is the main site for erythropoietin synthesis) (48). Anemia leads to insufficient oxygen supply to the body tissues, resulting in reduced ATP production by liver cells under hypoxic conditions, decreased activity of repair enzymes, slower regeneration of liver cells, and prolonged hospital stay (49). Platelets are the core indicators of the coagulation function (50). Patients with liver cancer experience complex changes in their coagulation system, which may make them more prone to bleeding or develop thrombotic complications (51). Thrombocytopenia significantly increases the risk of postoperative bleeding and may lead to an increase in the length of hospital stay. There have been only a few reports on the relationship between HALP and liver diseases. Some studies suggested that the HALP score can be used as an assessment tool for predicting the postoperative prognosis of patients with HCC (47, 52) and for evaluating the prognosis of HCC patients after liver transplantation (53, 54).

The CONUT, PNI, and HALP scores reflect abnormal nutritional, immune, hematopoietic and metabolic states from different aspects. The defined malnutrition may be related to the prolonged hospital stay of liver cancer patients after intervention treatment. This study has some limitations. Firstly, there are limitations in the research design. This study is a single-center retrospective study with a relatively small sample size, and there may be selection bias, which may not fully represent all the liver cancer intervention treatment populations. Future studies need to conduct multi-center prospective research to verify the results. Secondly, there are limitations in the timeliness of nutritional assessment. This study used laboratory indicators of interventional treatment for scoring, but did not dynamically monitor the changes in postoperative nutritional status, and could not assess the impact of nutritional intervention on hospital stay. Finally, there are limitations in the outcome indicators. The specific reasons for the prolonged hospital stay were not further analyzed (such as the type of complications, adjustment of treatment plans, and so on). Future studies can combine the occurrence of complications to deeply explore the causal chain between nutritional status and delayed recovery.

Future research can be conducted in the following directions. First, conduct prospective randomized controlled trials to verify the effectiveness of nutritional intervention in shortening the hospital stay after interventional treatment. Second, build a combined prediction model that includes nutritional scores, clinical characteristics, and imaging indicators to further improve the prediction accuracy of the risk of prolonged hospital stay. Third, explore the association between nutritional status and long-term postoperative prognosis (such as tumor recurrence and survival rate) to provide more comprehensive evidence for the optimization of comprehensive treatment strategies for liver cancer.

## Conclusion

This study confirmed that the malnutrition defined by the CONUT, PNI, and HALP scores was significantly associated with the prolonged hospital stay in liver cancer patients who underwent interventional treatment. Incorporating these readily accessible and cost-effective biomarkers into routine assessment protocols can enhance individualized risk stratification and guide clinical decision-making. These findings highlight the potential of immunonutritional and inflammatory markers to complement traditional morphological criteria in modern oncology.

## Data Availability

The original contributions presented in the study are included in the article/supplementary material. Further inquiries can be directed to the corresponding author.
